# Shape–color associations in an unrestricted color choice paradigm

**DOI:** 10.3389/fpsyg.2023.1129903

**Published:** 2023-06-02

**Authors:** Aurore Zelazny, Xun Liu, Thomas Alrik Sørensen

**Affiliations:** ^1^Centre for Cognitive Neuroscience, Department of Communication and Psychology, Aalborg University, Aalborg, Denmark; ^2^Sino-Danish College (SDC), University of Chinese Academy of Sciences, Beijing, China; ^3^Department of Psychology, University of the Chinese Academy of Sciences, Beijing, China

**Keywords:** shape–color associations, cross-modal correspondence, concept learning, ordinal concepts, shape learning

## Abstract

Since Kandinsky's claim for fundamental shape–color associations, several studies have revealed that those tendencies were not generalizable to the entire population and that different associations were more prevalent. Past studies, however, lacked a methodology that allowed participants to freely report their shape–color preferences. Here, we report data from 7,517 Danish individuals, using a free choice full color wheel for five different geometrical shapes. We find significant shape–hue associations for circle-red/yellow, triangle-green/yellow, square-blue, and pentagon/hexagon-magenta. The significant shape–hue associations are also more saturated than non-significant ones for the circle, triangle, and square. At the conceptual level, basic shapes, which show stronger associations, are linked to primary colors, and non-basic shapes to secondary colors. Shape-color associations seem indeed to follow the Berlin-Kay stages of entry into languages. This pattern had previously been described for graphemes and weekday–color associations. The methodology employed in our study can be repeated in different cultural contexts in the future. We also provide another instance of color associations for ordinal concepts that follow the stages of entry into languages.

## Introduction

Color perception begins with light waves entering the eye. Interestingly, in some cases, color perception can occur in the absence of color information entering the retina. Notably, when presented with black letters, individuals with grapheme–color synesthesia do experience colors (Cytowič, 1993; Ásgeirsson et al., 2015). Although synesthesia is a fascinating phenomenon, the general population seems to also make associations to categories across sensory modalities. Cross-modal correspondence [or association (Spence, 2011; Malfatti, 2014)] refers to this tendency for a large portion of the non-synesthete population to join features from different modalities together (Spence, 2011). For example, high-pitched sounds are commonly associated with brighter colors (Marks, 1987). Although sharing some aspects with synesthesia (e.g., mapping from one modality to another modality), the two phenomena differ on several accounts. Cross-modal correspondences are universal, with little variation across individuals, and unconscious (Deroy and Spence, 2013), contrary to synesthesia, where associations are idiosyncratic and conscious. Research on cross-modal correspondence became more and more prolific over the past couple of decades, with much interest geared toward auditory-visual correspondence [see Spence (2011) for a review]. Nevertheless, a large variety of modality pairs have been investigated, including smell–color (Gilbert et al., 1996; Demattè et al., 2006a), shape–smell (Hanson-Vaux et al., 2013), sound–taste (Knöferle and Spence, 2012), shape–taste (Velasco et al., 2015), taste–shape (Velasco et al., 2016), vision–touch (Martino and Marks, 2000), or smell–touch (Demattè et al., 2006b), to cite a few.

Among studies on color-related correspondences are those examining shape–color associations. These originate in the arts when Kandinsky (Kandinsky, 1912[1946]) famously postulated a fundamental link between shapes and colors, linking the triangle to yellow, the circle to blue, and the square to red. These associations were empirically examined using a priming paradigm (Kharkhurin, 2012) as well as with the Implicit Association Task (IAT; Makin and Wuerger, 2013). Both studies demonstrated that Kandinsky's original proposal is not generalizable to the entire population. Nevertheless, although Kandinsky's suggested associations were not supported, it is well-established that some shape-color associations are broadly available in the general population. Table 1 provides a summary of experimental results for the three shapes proposed by Kandinsky. These studies point to a general trend, namely, red/yellow circle, yellow/red triangle, and blue/red square. However, because these studies employed various paradigms, a direct comparison between them is challenging. Indeed, while some studies used an IAT paradigm (Makin and Wuerger, 2013; Chen et al., 2015b), others preferred a direct report method, i.e., asking participants to choose which color best fits various shape stimuli (Jacobsen, 2002; Jacobsen and Wolsdorff, 2007; Albertazzi et al., 2013; Chen et al., 2015a, 2016, 2019; Dreksler and Spence, 2019; Hanada, 2019). Comparison between these direct report studies is also difficult, given that they differed in the number of color options they offered as answers to their participants. The most restricted ones (Jacobsen, 2002; Jacobsen and Wolsdorff, 2007) only offered three possible color answers (red, yellow, and blue), and each of them could only be used once. Other studies (Chen et al., 2016, 2019) instead offered four color chips (red, yellow, blue, and green), which could be reused for several shapes. Hanada (2019) proposed 15 color chips (light blue, blue, cyan, green, light green, mint green, light yellow, yellow, orange, pink, red, magenta, purple, violet, and brown) consisting of eight Berlin–Kay color categories (Berlin and Kay, 1969), plus seven more precise colors, to bring variety. Dreksler and Spence (2019) selected 36 color chips at maximum saturation, consisting of 10 color categories featuring eight Berlin–Kay color categories (Berlin and Kay, 1969), plus two more varieties of blue (red, orange, yellow, green, aquamarine, sky, blue, purple, pink, and brown) in three different levels of lightness, plus a 6-step grayscale. Finally, Albertazzi et al. (2013) and Chen et al. (2015a) used 40 preselected color chips, taken from the Natural Color System (NCS) at maximum saturation.

**Table 1:** Summary of findings from previous shape–color association studies, compared to Kandinsky's proposal for the circle, triangle, and square.

**References**	**Nationality**	***N***	**○**	**△**	**□**
Kandinsky (1912[1946])	Russian	1	B	Y	R
Jacobsen (2002)	German	200	Y	R	B
Jacobsen and Wolsdorff (2007)	German	74	R	Y	B
Kharkhurin (2012)	United Arab Emirates	284	NA	NA	B
Albertazzi et al. (2013)	Italian	60	R, Y	Y	R, B
Makin and Wuerger (2013)	British	36	NA	NA	NA
Chen et al. (2015a)	Japanese	138	R, Y, O	Y	B
Chen et al. (2015b)	Japanese	38	R	Y	B
Chen et al. (2016)	Japanese	91 + 95	R	Y	B, G
Dreksler and Spence (2019)	International	64	R, Y	R, Y	NA
Hanada (2019)	Japanese	50	R	Y	NA
Chen et al. (2019)	Chinese	99 + 117	R	Y	B

B: blue; G: green; O: orange; R: red; Y: yellow; NA: non-significant/non-tested association.

The use of pre-selected color chips, although facilitating data analysis, may in some cases have limited and potentially influenced the responses from the participants. In the case of three color options where participants were not allowed to reuse a color chip for two different shapes (Jacobsen, 2002; Jacobsen and Wolsdorff, 2007), it is possible that participants found themselves forced to report a shape–color association they did not experience due to the one-to-one pairing restriction.

The color chips proposed by Albertazzi et al. (2013) and Chen et al. (2015a) were based on the 40 hues of the NCS at maximum saturation, which failed to provide color categories such as cyan, brown, or pink. On the other hand, Dreksler and Spence (2019) provided a restricted version of the color wheel, covering the eight Berlin–Kay hue categories, plus a grayscale. It contained, however, an over-representation of blue chips (nine chips), compared to other color categories (three chips each). Furthermore, the provided chips had not been controlled for perceptual differences, resulting in chips, within and across hues, that are close to perceptual similarity (Robertson, 1977).

Colors are the sum of three components: hue, lightness, and saturation. Hue is the aspect that we commonly call “color”, such as red, blue, or green. Lightness refers to the amount of white or black in the color, while saturation refers to the degree of colorfulness of the hue, i.e., how much gray is contained in the color. In recent studies (Albertazzi et al., 2013; Chen et al., 2015a; Dreksler and Spence, 2019), the saturation aspect of colors was set to maximum, which did not allow the evaluation of the involvement of saturation in shape–color associations. Saturation and lightness seem though to play a role in cross-modal correspondences, as they were indeed found to highly influence the color-emotion associations (Schloss et al., 2020), saturation levels were related to haptic stimuli intensity (Slobodenyuk et al., 2015) and saturation differences reflected increases in loudness and frequency in sound-color associations (Hamilton-Fletcher et al., 2017).

Several explanations for the specific shape–color associations have been proposed. Shape–color associations have been hypothesized to be mediated by perceived temperatures, where shapes rated warmer were associated with warm colors, such as red and yellow, whereas shapes regarded as cold align more with the color blue (Albertazzi et al., 2013; Chen et al., 2015a). Malfatti et al. (2014), however, also found that emotions could as well mediate the shape–color associations, with angry shapes being associated with angry colors.

Prior research on color-related associations has found a tendency for ordinal concepts to be non-randomly associated with colors following Berlin and Kay (1969)'s six stages of entry into languages of color terms. Berlin and Kay (1969) established that color terms appear in languages following a precise order. Languages that only have two color terms will therefore feature words meaning “black” and “white”, but languages that have three terms will have words that designate the colors “black”, “white” and “red”. Berlin and Kay (1969) managed thus to define six stages of entry into languages for color terms.

Ordinal concepts, such as numbers and days of the week (Shanon, 1982), as well as numbers and letters in both English and Arabic (van Leeuwen et al., 2016), were found to be associated with colors that follow the six stages defined by Berlin and Kay (1969). These studies thus demonstrate the involvement of higher-level concept mapping in color associations.

Previous shape–color studies did not allow for investigation of this question, as intrinsic order was not possible to be derived from the stimuli list. To test this hypothesis, we selected five shapes, with increasing complexity, as denoted by their number of sides (circle, triangle, square, hexagon, and pentagon). The order is even more clear for our tested Danish population as the shape names are directly derived from the number of sides they contain [trekant (“three-edge”), firekant (“four-edge”), femkant (“five-edge”), and sekskant (“six-edge”)].

To investigate the shape–color associations in a direct report paradigm where we want to avoid the potential biases cited above, we performed a large-scale data collection in collaboration with the Trapholt Museum in Denmark (Zelazny and Sørensen, 2020), using a full color wheel inspired by work on synesthesia (Ásgeirsson et al., 2015) and work on color-related cross-modal associations involving various sensory modalities such as haptic (Lindborg, 2014; Slobodenyuk et al., 2015; Delazio et al., 2017) and sound (Hamilton-Fletcher et al., 2017). Museum visitors were invited to freely associate five pre-defined shapes (circle, triangle, square, hexagon, and pentagon) with the color they feel fits best. The full color spectrum, including the option to modify both lightness and saturation, was available for the participants. This method allows for a more thorough and genuine investigation of shape–color association tendencies without placing any restrictions on the participants' access to colors.

## Methods

The study has been approved by Aalborg University and complies with the GDPR rules. The study falls under the Danish National Videnskabsetisk Komité Law (§ 14, part 2), by which behavioral studies not involving biological material are exempted from the regional ethics committee. The study was performed in accordance with the ethical standards of the Declaration of Helsinki (1964) and its subsequent amendments. Participants gave written informed consent to data collection.

### Apparatus and stimuli

The experiment was part of a set-up containing four experiments, which were independent from each other. Only the results from one of the four experiments are used in the present study. The setup was designed to be self-explanatory and to be able to function without any supervision. The experiment was run on a Windows 10 Lenovo Miix 320 (10.1-inch screen) two-in-one computer, consisting of an FHD screen with a 1920 × 1200 resolution and a detachable keyboard, used in touchscreen mode. The set of experiments was run through a custom-made webpage, using Chrome in kiosk mode, coded using the Django web framework.

**Figure 1:**
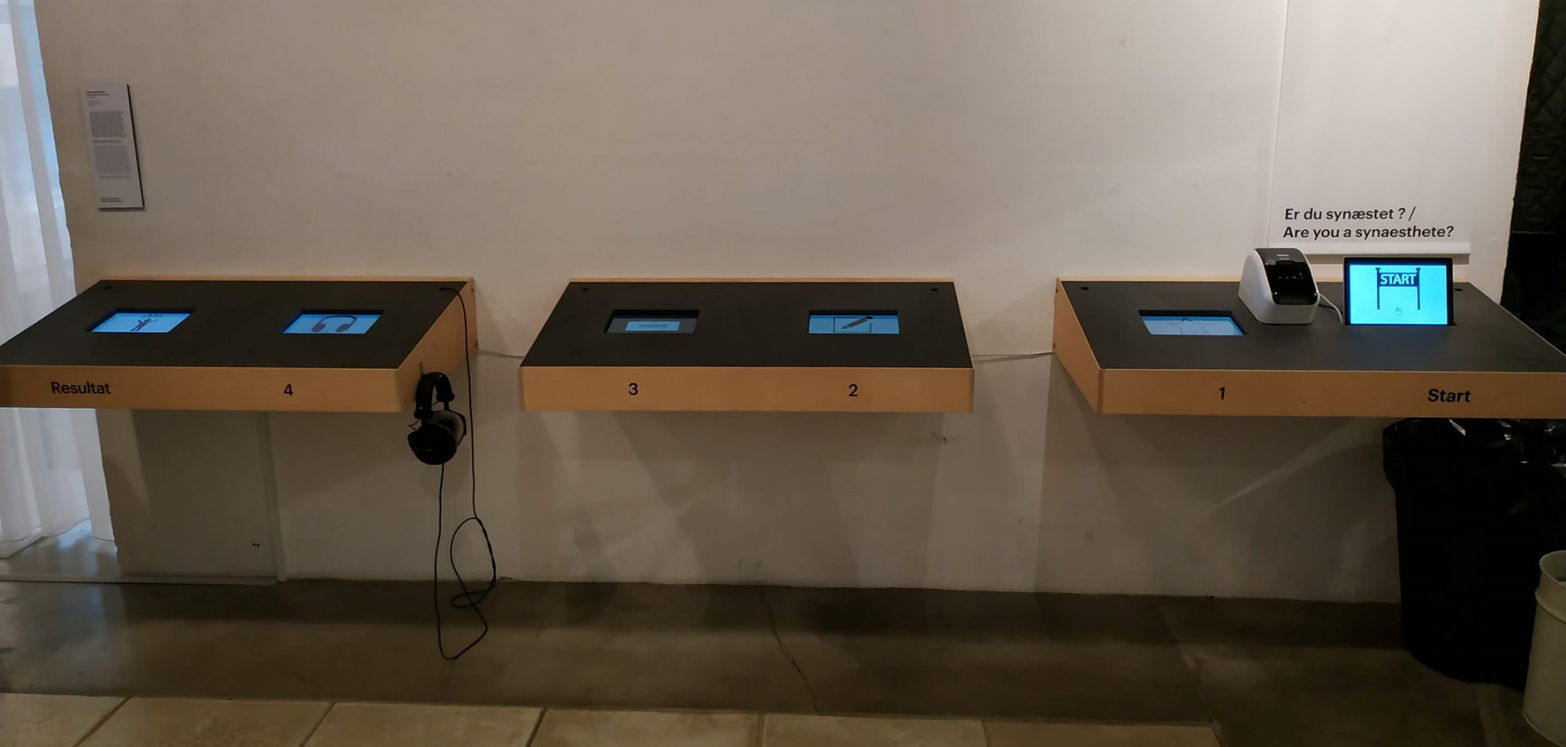
Experimental setup at the Trapholt Museum, Denmark. The experiment was part of a 4-experiment setup. The experiment described in this study was conducted at the station marked “1.” Visitors received a unique ID number at the “Start” station and could review their responses at the “Result” station.

Three custom-made wooden frames were used to secure the computers in place and were attached to an adjacent wall, ~ 90 cm from the floor. The wooden frames were aligned along the wall, 20 cm apart. The setup consisted of a total of six computers, each wooden frame hosting two computers. A printer was also used, which was fixated between the two computers from the rightmost wooden frame (Figure 1).

**Figure 2:**
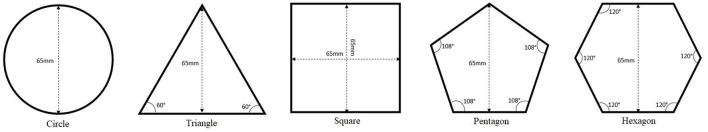
Five shapes used as stimuli.

**Figure 3:**
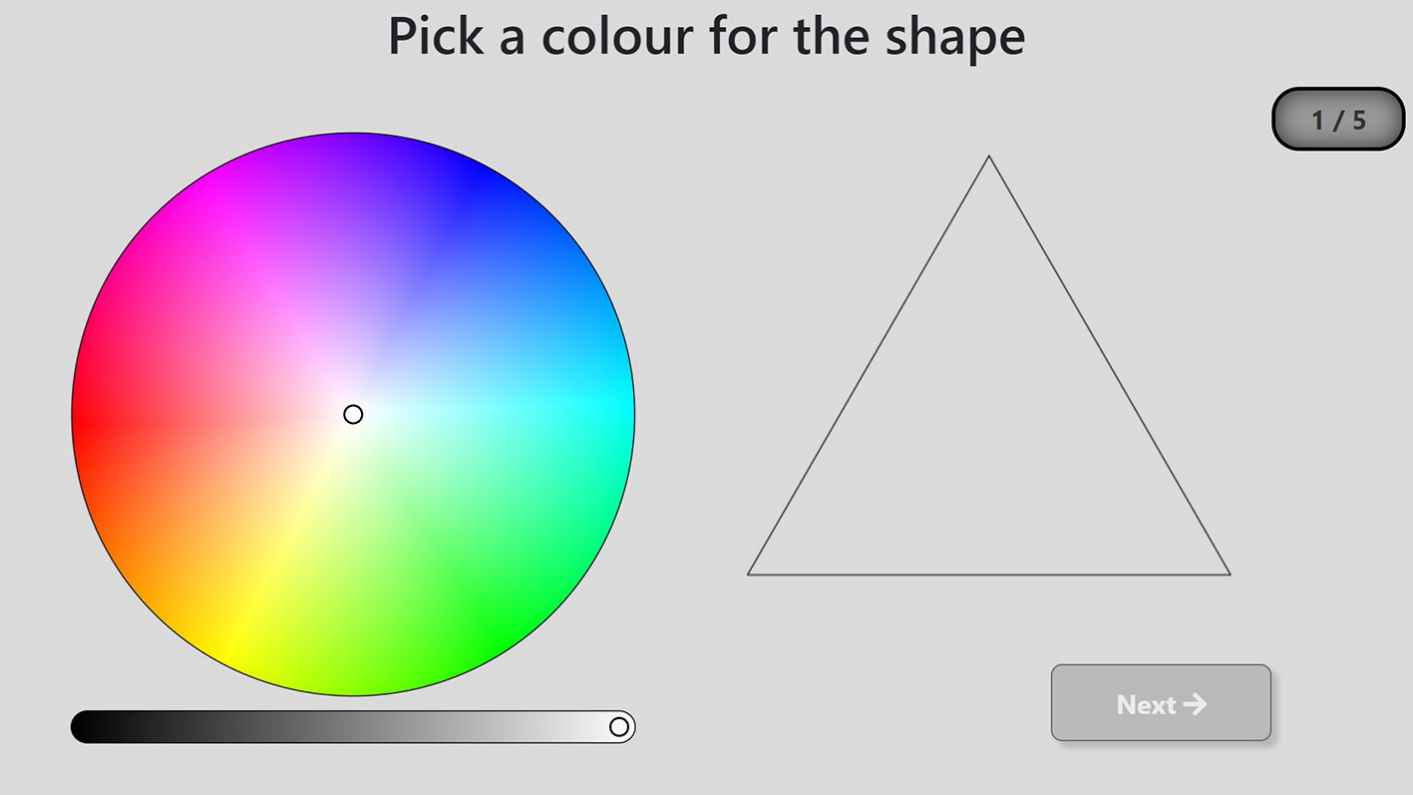
Screenshot of an experimental trial.

Viewing distance could not precisely be controlled for, therefore display dimensions will be described in millimeters rather than in visual angle degrees. The stimuli consisted of five two-dimensional shapes (circle, triangle, square, pentagon, and hexagon) against a gray background [RGB (219, 219, 219), 266 cd/m^2^]. Each of the shapes was dimensioned as shown in Figure 2. The shapes were presented one after the other on the right-hand side of the screen and a full color wheel of 85 mm × 85 mm was displayed on the left-hand side. A lightness bar of 87 mm × 5 mm was positioned just below the color wheel. The color wheel and shape stimuli were separated by a 33-mm gap. Upon presentation, the outline of the shape stimulus was shown using a one-pixel black line and transparent filling. As soon as an area of the color wheel was touched, the shape was automatically filled in with the corresponding color and the black outline disappeared. Unlimited color changes were possible, and there was no time limit to respond. Once satisfied with the selected color, the participants had to press a 33 mm × 12 mm “Next” button displayed at the bottom right of the screen to see the next shape. A 20 mm × 10 mm counter was also inserted at the top right of the screen to maintain engagement. See Figure 3 for a screenshot of the experimental layout. Each shape was shown only once, and the presentation was randomized, resulting in five trials per participant. Only the CIELab values [i.e., the coordinates of the colors in the CIELab color space (International Organization for Standardization, 2018)] of the last selected color (i.e., the one displayed on the shape before pressing the “Next” button) were collected for each shape for each participant.

### Procedure

The data collection was performed at the Trapholt Museum (located in Kolding, Denmark) as part of the SENSEME exhibit, inviting visitors to explore their multisensory perceptions. The visitors could not be isolated to perform the experiment, nevertheless, particular attention was made to not place the setup close to any decoration or art elements that could remind the participants of any of the stimuli presented. As such, the setup was placed in a quiet area of the museum, facing a white wall to avoid distractions (see Figure 1). The setup was accessible without having to go through the various exhibition rooms and the experiment could also be performed as a first stop before entering the exhibition rooms. Furthermore, none of the art pieces presented at the SENSEME exhibit featured sensory responses to shapes and focused rather on multisensory responses to taste, touch, movement, and music.

The experiment reported here was part of a four-experiment set-up, the other three experiments asked participants to report their color experience to a set of nine graphemes (i.e., 1, 4, 8, A, D, N, 水, 竹, and 血), to report their color experience to seven different pure tones (i.e., ranging from C4 to B4) and to report their spatial representation of a year. The experiments could be performed in any order; nevertheless, the shape–color association experiment being the one placed the closest to the “Start” station (Figure 1, see below a description of data collected at the Start station), 68% of the visitors completed this experiment before any of the three others. Each experiment could be performed only once by each participant.

The museum context for data collection comes with some limitations. The experimental paradigm had to be brief to ensure that the participants completed the task. For this reason, we tried to keep the testing time to <5 min in total, to lower the dropout rate. Similarly, we only tested five shapes to remain within the time constraints. The number of shapes was determined based on the maximum time estimated to provide a color answer for each stimulus using a color wheel.

The museum's floor plan did not allow us to isolate the visitors during testing. Paradigms based on fast stimuli presentation and requiring an isolated environment were avoided, such as experiments based on priming or implicit experience. Direct measures, where the participants were asked to report their experience, were therefore used here. For the reasons cited above, no time limit was introduced for participants to deliver their responses either.

The visitors were provided with a unique ID number after having completed a demographic questionnaire (Station marked as “Start” in Figure 1) and could also select their preferred language (English or Danish). Upon entering their ID number to start the “shape-color” experiment (Station marked as “1” in Figure 1), an instruction screen appeared. It explained the task (“*Pick the color that you feel fits each shape best, by pressing on the color wheel. When you are satisfied with the chosen color, press Next to see the next shape*”) and featured a gif animation on how to perform it, using an illustrating example, a star, which was not used as a stimulus during the test. A “?” button was also there to provide a detailed step-by-step procedure. Upon pressing the “Begin” button, the visitors were brought to the experiment screen, starting the first of five trials.

After the test was completed, the visitors received some feedback, to try to maintain engagement in the experiments and lower the drop-off rate throughout the whole setup. The feedback consisted of a single screen featuring the five shapes each displayed in the color chosen by the visitor, along with reference results collected during the piloting session. The feedback was provided after task completion to avoid biasing the reports.

### Participants

A total of 8,841 visitors completed the task. The data from 106 visitors presented issues (trials were not forwarded to the database) and were removed. An additional 196 participants were removed due to having reported colorblindness. Abnormal and not corrected-to-normal visual acuity were reported by 195 visitors, who were also removed from the analysis. Of the remaining 8,344 visitors, 7,550 reported to be of Danish nationality. Among those, 32 were removed due to reporting an age below 6 years, reflecting potentially a typo or parents performing the task for their child, and one was removed for reporting an aberrant age (i.e., 900 years). This resulted in a total of 7,517 visitors remaining for data analysis and comprised 5,186 women (mean age = 37.1; SD = 19.7; range: 6–89) and 2,331 men (mean age = 36; SD = 21.2; range: 6–100). Among the 7,517 visitors, a total of 993 reported having some type of synesthesia. These were nevertheless included in the analysis, as we could not formally validate their synesthesia and did not inquire about the type of synesthesia they had.

## Results

The CIELab values allow measuring the three components of color: lightness, saturation, and hue. The CIELab color space is built around three axes: L, *a*, and *b*. Lightness is denoted by the value along the L axis. The *a* and *b* axes represent continua of colors ranging from green to red and blue to yellow, respectively. Saturation and hue are retrieved by converting the cartesian coordinates denoted by the *a* and *b* axes to polar coordinates, where the saturation is defined as the length *S* of the projection along the *a* and *b* axes, calculated using formula (1) and hue as the angle *H* relative to the positive pole of the *a* axis, calculated using formula (2).


(1) $$\begin{array}{l} S\;=\;\sqrt{a^{2}+b^{2}} \end{array}$$

(2) $$\begin{array}{l} H\;=\;\tan^{-1}\frac{b}{a} \end{array}$$

### Hue analysis

**Figure 4:**
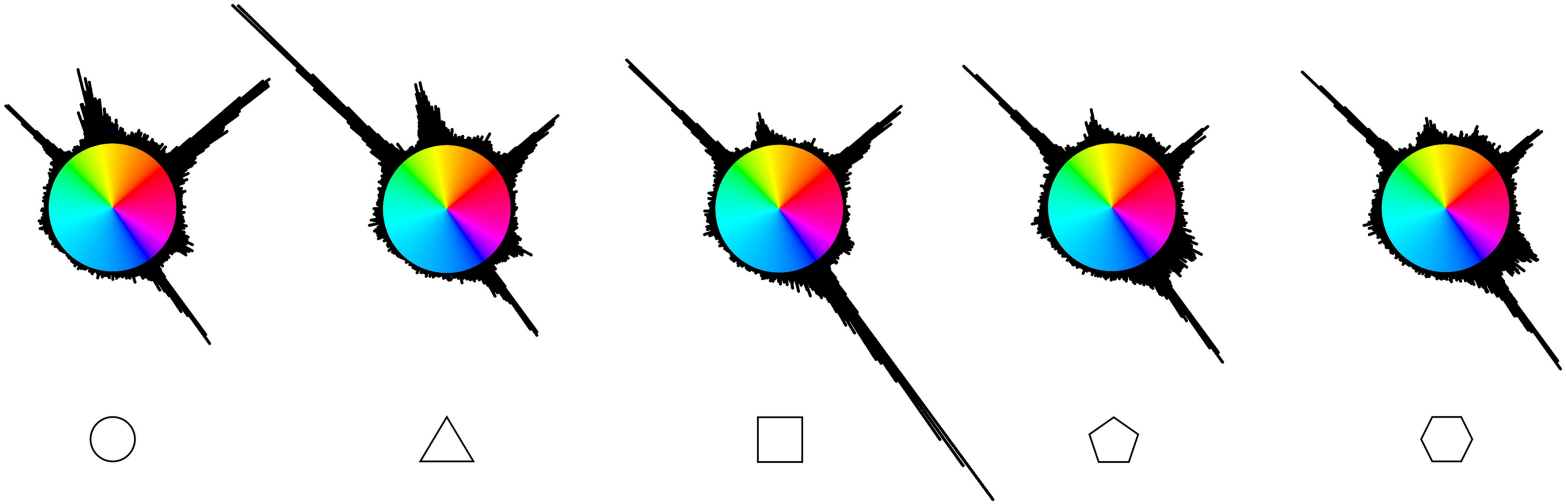
Cumulative responses for each shape along the a and b coordinates of the CIELab space. The color of each bin is represented at maximum saturation. *N* = 7,517.

**Figure 5:**
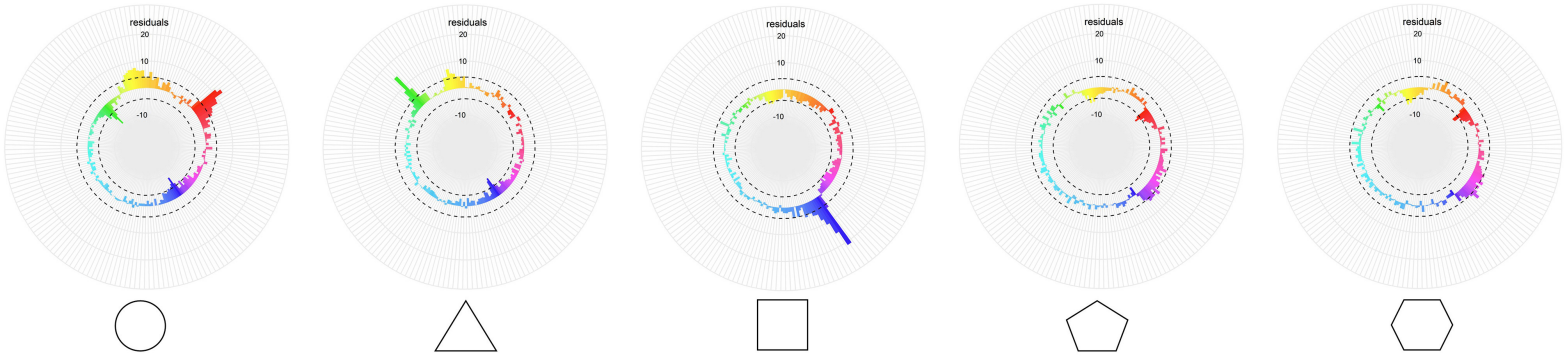
*X*^2^-residuals for each hue bin and shape. The dashed line depicts the Bonferroni-corrected significance threshold. The colors displayed are the average color for each of the hue bins for the whole dataset.

The participants' answers were not evenly distributed along the hue wheel (Figure 4), with some hues concentrating larger portions of answers than others. To determine what areas of the hue wheel were more often associated with each of the five shapes, the CIELab space was divided into 180 bins of 2° angles each. Responses with a saturation value below 1 were excluded, as they denote a grayscale response. These represented 326 responses (0.87% of the data). A chi-square test of independence was carried out on the frequency of each bin for each shape (5 × 180) to examine shape–hue association tendencies. The chi-square test revealed a significant association between hue bins and shapes [χ^2^(716, *N* = 37,259) = 4937.7, *p* < 0.001, ϕ _C_ = 0.18, CI_.95_ (0.163, 0.174)]. *Post-hoc* residual analysis, Bonferroni-corrected to the threshold *z* > 4.03 at *p* < 0.05 (Figure 5), showed that 50° to 63° hues [red hues; peaking at 52° (*z* = 12.7)], as well as 340° to 3°, plus 8° and 9° hues [yellow; peaking at 350° (*z* = 7.8)], were significantly more chosen for the circle. Hues corresponding to 310° to 317° [green; peaking at 314° (*z* = 14.5)] and 342° to 359° [yellow; peaking at 346° (*z* = 7.6)] were significantly more chosen for the triangle, and hues corresponding to 142° to 162°, plus 166°, 167°, 170° and 171° [blue; peaking at 144° (*z* = 21.7)] for the square. The pentagon was significantly more associated with hues corresponding to 128° and 129°, and 132° to 139° [magenta/purple; peaking at 128° (*z* = 5.7) and 136° (*z* = 5.3)]. The hexagon was significantly more associated with hues corresponding to 22° and 23° [orange (*z* = 4.2)], 114° and 115°, 118° to 121°, 124° and 125°, 128° to 133° [magenta/purple; peaking at 120° (*z* = 5.2) and 130° (*z* = 6.9)].

### Saturation analysis

Maximum saturation is not denoted by the same *S* value for every hue angle in the CIELab space (e.g., full saturation at a 255° hue angle (cyan) corresponds to a *S* value of 40, while at a 143° hue angle (blue), it corresponds to a *S* value of 133.8). To make sure that the saturation analysis was not confounded by saturation differences across hues, participant responses' *S* values were normalized to a 0–100 scale, where 0 denotes a *S* value of 0 (grayscale) and 100 the maximum saturation value possible at a given hue angle.

A one-way repeated measures ANOVA was run on the normalized saturation, as a function of shape. A significant main effect of shape was found, *F*(1, 3.85) = 21.58, *p* < 0.001, $\eta_{\text{p}}{}^{2}$ = 0.003.

*Post-hoc* Bonferroni-corrected paired-sample *t*-tests were run to compare normalized saturation between shapes. The triangle was found to have a significantly higher saturation (M_T_ = 85.2; SD_T_ = 16.8) than all four other shapes (M_C_ = 83.5; SD_C_ = 20.2; M_S_ = 83.7; SD_S_ = 20.5; M_P_ = 83.3; SD_P_ = 17.9; M_H_ = 83.1; SD_H_ = 18.4), all *p*s <0.001.

This tendency for the triangle to be associated with more saturated colors may be because the hues significantly associated with the triangle (310° to 317° and 342° to 359°) have a higher saturation. To test this, the data were split into four groups, whether the data point corresponds to a triangle trial or not (shape type: 2 levels) and whether the data point falls within the hues significantly associated with the triangle (310° to 317° and 342° to 359°) or not (hue bin: 2 levels). A two-by-two ANOVA was run on the normalized saturation. A main effect of shape type was found, *F*(1, 37581) = 29.87, *p* < 0.001, $\eta_{\text{p}}{}^{2}$ ≤ 0.001, by which the triangle was more saturated (M = 85.2; SD = 16.8) than the other shapes (M = 83.4; SD = 19.3), as well as a main effect of hue bin was found, *F*(1, 37581) = 206.57, *p* < 0.001, $\eta_{\text{p}}{}^{2}$ = 0.005, showing that the bins 310°-317° and 342°-359° are significantly more saturated (M = 86.7; SD = 14.7) than all other bins (M = 83; SD = 19.7). A significant interaction of shape type and hue bin was also found, *F*(1, 37581) = 4.27, *p* < 0.001, $\eta_{\text{p}}{}^{2}$ <= 0.001. *Post-hoc* Bonferroni-corrected pairwise comparison was run on the two levels of the shape type condition and on the two levels of the hue bin condition to investigate the interaction. All comparisons were significant (significant hue bins for the triangle: M = 88.2; SD = 12.8; non-significant hue bins for the triangle: M = 83.9; SD = 18.2; significant hue bins for the other shapes: M = 86.1; SD = 15.4; non-significant hue bins for the other shapes: M = 82.9; SD = 19.9), with all *p*s <0.001.

The hues significantly associated with the triangle (310°-317° and 342°-359°) tended to be more saturated overall, but the effect was even more present when those hues were picked for the triangle, compared to the other shapes.

### Shape–hue associations and saturation interaction

**Figure 6:**
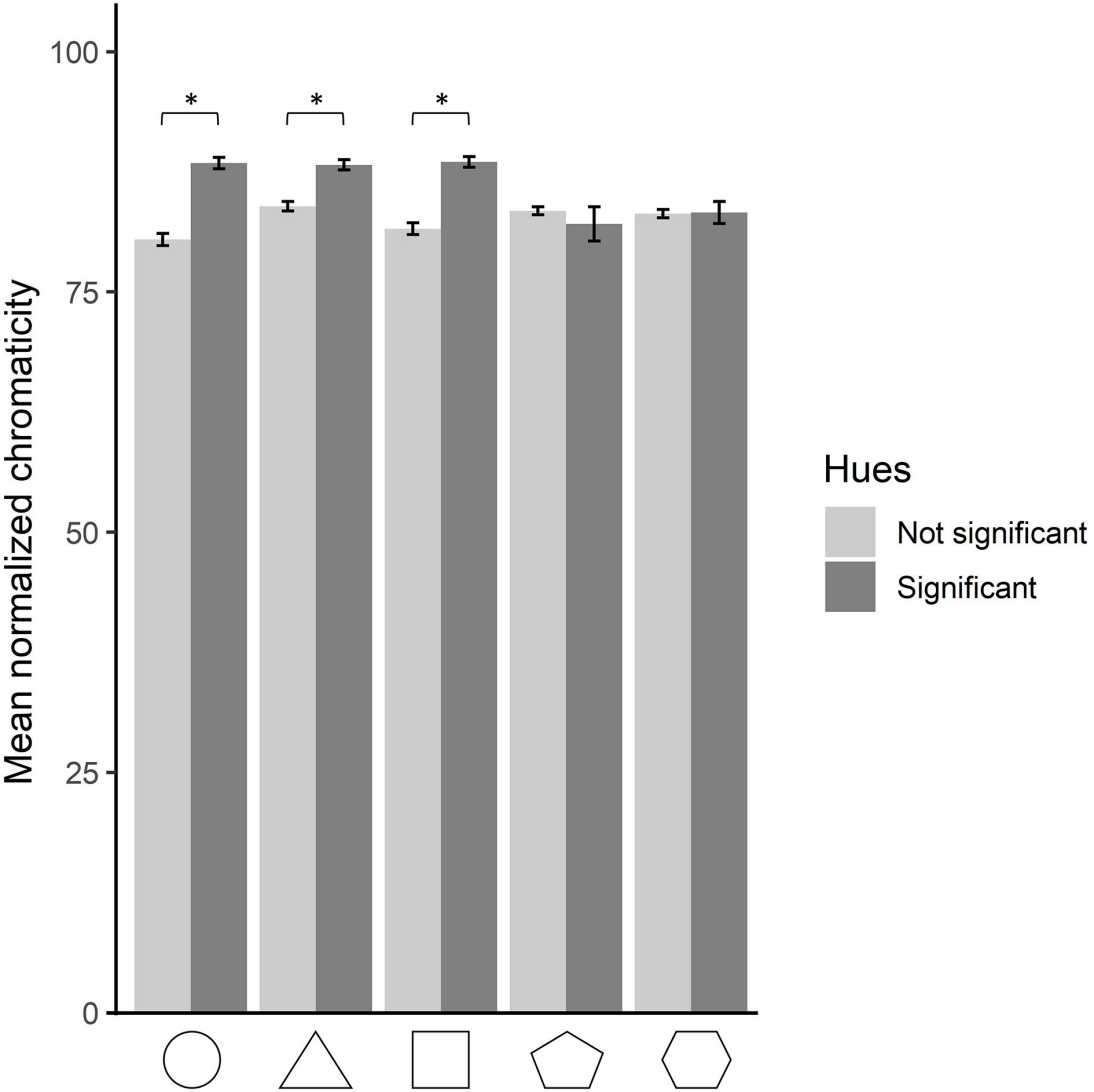
Mean normalized saturation for each shape, for hues resulting significant and non-significant from the hue analysis. The error bars depict 95% confidence intervals. Statistically significant comparisons at *p* < 0.05 are marked by an asterisk.

As shown above, the yellow and green hues significantly associated with the triangle were more saturated when chosen as a response to the triangle than when chosen as a response to the other shapes. This raises the question of whether significant shape–hue associations are more saturated than non-significant ones for the four other shapes as well. To test this, each data point was marked as either being a choice that agrees with the significant shape–hue associations of the population or not. A two-by-two ANOVA was run on the normalized saturation as a function of shape (five levels) and significance (two levels). A main effect of significance was found, *F*(1, 37575) = 482.64, *p* < 0.001, $\eta_{\text{p}}{}^{2}$ = 0.013, indicating that significant shape–hue associations are more saturated than non-significant ones (significant associations: M = 87.6; SD = 14.9; non-significant associations: M = 82.6; SD = 19.7). A significant interaction of significance and shape was also found, *F*(4, 37575) = 38.63, *p* < 0.001, $\eta_{\text{p}}{}^{2}$ = 0.004. *Post-hoc* Bonferroni-corrected pairwise comparison was run on the two levels of the significance condition for each of the five shapes. Comparisons were significant for the circle, triangle, and square, showing that they were more saturated in their significant hues than in the non-significant ones, with all *p*s <0.001 (Figure 6). No difference was found, however, for the pentagon and hexagon.

### Lightness analysis

A one-way repeated measures ANOVA was run on the L value of the CIELab space, as a function of shape. A significant main effect of shape was found, *F*(3.94, 29633.17) = 281.03, *p* < 0.001, $\eta_{\text{p}}{}^{2}$ = 0.036. *Post-hoc* Bonferroni-corrected paired-sample *t*-tests were run to compare lightness values between shapes. The lightness values of all shapes were significantly different from each other, except for the pentagon and hexagon (M_C_ = 69.2; SD_C_ = 20.1; M_T_ = 71.7; SD_T_ = 19.9; M_S_ = 61.2; SD_S_ = 22.2; M_P_ = 66.5; SD_P_ = 19.9; M_H_ = 66.4; SD_H_ = 19.8), all *p*s <0.001. The triangle appears therefore as the shape associated with the highest lightness value, followed by the circle. The pentagon and hexagon both share a lower lightness value than the circle. Finally, the square is the shape associated with the lowest lightness value.

### Shape–hue associations and lightness interaction

**Figure 7:**
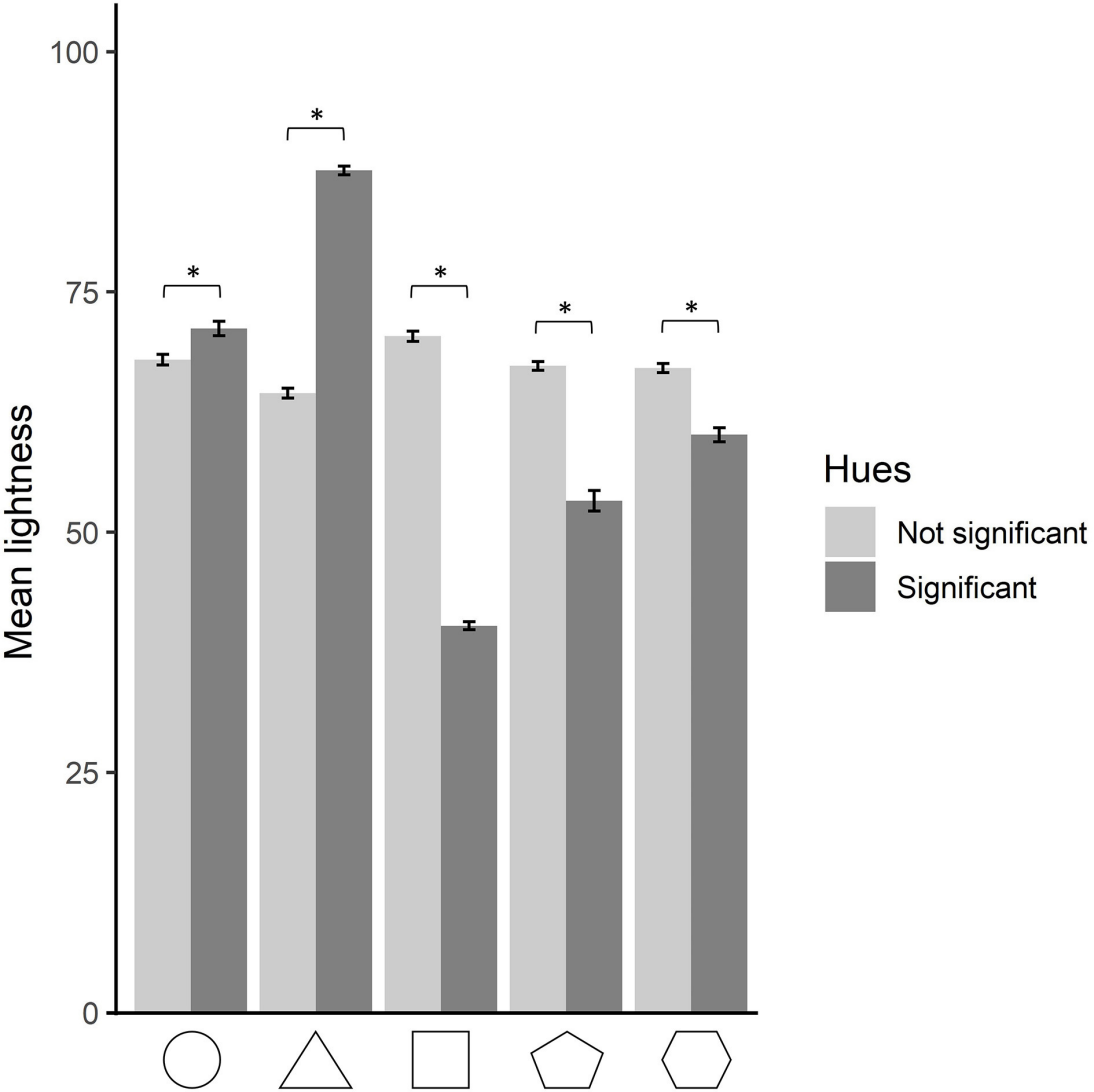
Mean lightness for each shape, for hues resulting significant and non-significant from the hue analysis. The error bars depict 95% confidence intervals. Statistically significant comparisons at *p* < 0.05 are marked by an asterisk.

Following our finding on saturation differing between significant and non-significant shape–hue associations, a similar analysis was run regarding lightness. The goal was to compare whether the lightness differs in significant shape–hue associations compared to non-significant ones, for each shape separately. A two-by-two ANOVA was run on the lightness value as a function of shape and significance. A main effect of significance was found, *F*(1, 37575) = 103.98, *p* < 0.001, $\eta_{\text{p}}{}^{2}$ = 0.003, indicating that non-significant shape–hue associations are brighter than significant ones (significant associations: M = 65.7; SD = 23.3; non-significant associations: M = 67.4; SD = 19.8). A significant interaction of significance and shape was found, *F*(4, 37575) = 1710.38, *p* < 0.001, $\eta_{\text{p}}{}^{2}$ = 0.154. *Post-hoc* Bonferroni-corrected pairwise comparison was run on the two levels of the significance condition for each of the five shapes. All comparisons were significant, with the circle and triangle being brighter in their significant hues than in the non-significant ones. Following a reversed pattern, the square, pentagon, and hexagon were less bright in their significant hues than in the non-significant ones, with all *p*s <0.001 (Figure 7).

### Color analysis

The three components of color (i.e., lightness, saturation, and hue) were this time considered together. The goal was to evaluate what areas of the color space were more often chosen for each shape. A density-based spatial cluster analysis was carried out using the DBSCAN algorithm (Ester et al., 1996). The technique searches for areas of high-density points, which come to form clusters, and leaves out outliers that occur in low-density areas. This method was favored over the k-mean algorithm, which relies on Euclidean distances for forming clusters, as a method to account for noise in the data. The two main parameters of the DBSCAN algorithm are the epsilon, which defines the minimum distance between two clusters, and minPts, which defines the minimum number of neighbors needed for cluster identification.

**Figure 8:**
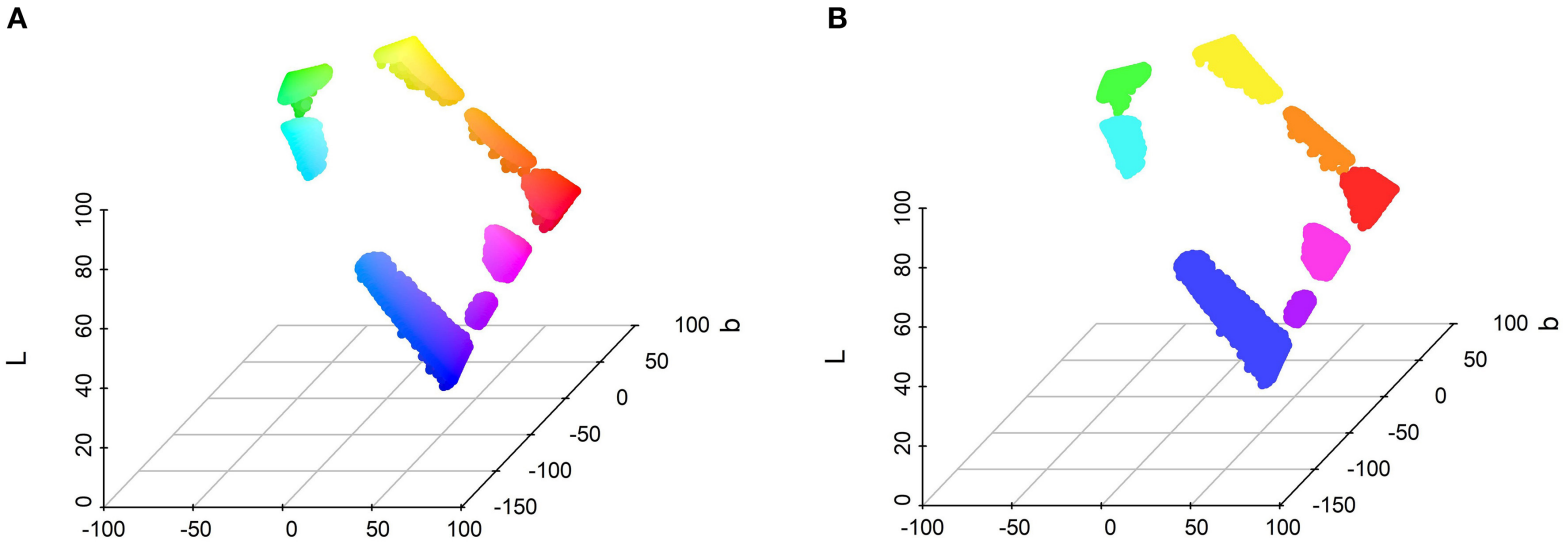
Positions in the CIELab space of the eight clusters from the analysis run on the whole dataset. **(A)** The color depicted corresponds to the corresponding color in the CIELab space. **(B)** The color depicted corresponds to the average color of each cluster.

In the present study, six clustering analyses were performed: one for each shape, to identify whether the number and location of high-density areas vary between shapes, and one for the data as a whole, which then serves as a basis for further analysis (chi-square test of independence). For the five shapes analyses, the epsilon parameter was set at 6 and the minPts parameter at 50. For the whole data analysis, the epsilon parameter was set at 6 and the minPts parameter at 250. The mean CIElab values of the resulting clusters for the whole data analysis can be found in Table 2 and the disposition and colors of the data points are shown in Figure 8. The clusters resulting from the clustering analysis run on each of the five shapes independently are gathered in Supplementary Table S1 of the Supplementary material.

**Table 2:** Mean CIELab coordinate values for the eight clusters resulting from the whole-data analysis, along with their frequency count and color name.

**Cluster**	**L**	**a**	**b**	**Color name**	**Frequency**
1	42.09	57.96	−91.46	Blue	6,132
2	88.53	−77.97	72.75	Green	4,135
3	55	75.33	55.22	Red	4,788
4	89.65	−43.87	−11.45	Cyan	1,552
5	93.23	−15.9	85.19	Yellow	3,555
6	61.46	86.97	−46.92	Magenta	1,954
7	70.03	36.07	70.11	Orange	1,327
8	48.89	84.5	−79.89	Purple	312

### Shape–cluster associations

A chi-square test of independence was carried out on the frequency of the clusters resulting from the whole-data analysis for each shape (5 × 8) to examine shape–cluster association tendencies. The chi-square test revealed a significant association between clusters and shapes ([(28, N = 23,755) = 3139.4, *p* < 0.001, ϕ _C_ = 0.18, CI_.95_ (0.175, 0.187)].

**Figure 9:**
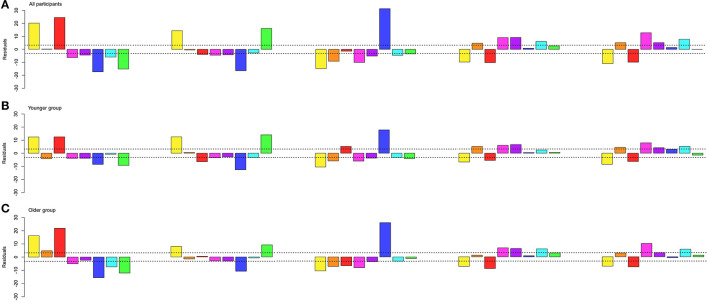
*X*^2^-residuals for each cluster from the whole dataset and for each shape. The dash line depicts the Bonferroni-corrected significant threshold. The colors displayed are the average color for each cluster for the whole dataset. **(A)** Residuals for the analysis of all participants. **(B)** Residuals for the analysis for the 6- to 34-year-old group (younger group). **(C)** Residuals for the analysis for the 35- to 100-year-old group (older group).

*Post-hoc* residual analysis, Bonferroni-corrected to the threshold, *z* > 3.23 at *p* < 0.05 (Figure 9, top panel), showed that the circle was significantly more chosen for cluster 3 (red; *z* = 24.5) and cluster 5 (yellow; *z* = 20.2). Cluster 2 (green; *z* = 16.1) and cluster 5 (yellow; *z* = 14.4) were significantly more chosen for the triangle, and cluster 1 (blue; *z* = 31.3) for the square. The pentagon was significantly more associated with cluster 4 (cyan; *z* = 6.2), cluster 6 (magenta; *z* = 9), cluster 7 (orange; *z* = 4.7), and cluster 8 (purple; *z* = 9.2), as well as the hexagon with cluster 4 (cyan; *z* = 7.8), cluster 6 (magenta; *z* = 12.8), cluster 7 (orange; *z* = 5.2), and cluster 8 (purple; *z* = 5.2).

### Shape–cluster associations per age group

The age distribution of our group of participants follows a bimodal distribution, with a first group (younger group) ranging from 6 to 34 years of age (*N* = 3,637), and a second group (older group) ranging from 35 to 100 years of age (*N* = 3,880). Differences in shape–color associations found between studies conducted in Germany were imputed to sub-cultural differences, namely participants were on average 20 years younger (median age: 23 years; range: 17–32 years) in an early study (Jacobsen, 2002) compared to participants from a later study (mean age: 43 years, range: 23–63 years) (Jacobsen and Wolsdorff, 2007). In both studies, the square was associated with blue, but the younger participant group associated the circle with yellow and the triangle with red (Jacobsen, 2002), while the older participant group followed an opposite pattern, by associating the circle with red and the triangle with yellow (Jacobsen and Wolsdorff, 2007).

A log-linear analysis (Christensen, 1997) with a Poisson model was carried out on the frequency of the cluster for each shape for each age group. The three-way log-linear analysis of shape (5) × cluster (8) × group (2) revealed an interaction of shape, cluster, and age group [χ^2^(28, *N* = 23,755) = 192.6, *p* < 0.001].

A *post-hoc* chi-square test of independence on the frequency of each cluster (8) for each shape (5) was run for each age group (2) to examine significant shape–color associations for different age ranges. The results came out as significant for both age groups [Younger: χ^2^(28, *N* = 10,652) = 1388.4, *p* < 0.001, ϕ _C_ = 0.18, CI_.95_ (0.169, 0.188); Older: χ^2^(28, *N* = 13,103) = 1952.7, *p* < 0.001, ϕ _C_ = 0.193, CI_.95_ (0.183, 0.2)]. A Bonferroni-corrected threshold, *z* > 3.23 at *p* < 0.05, revealed that different associations between groups (Figure 9, middle and bottom panels) occurred for the circle regarding the orange association in the older group (*z* = 4.78), for the square with significant associations to red for the younger group (*z* = 5.13), for the pentagon with significant associations to orange (*z* = 5.2) for the younger group and cyan (*z* = 6.09) for the older group, and for the hexagon with significant associations to purple (*z* = 4.24) for the younger group. All other shape–color associations did not significantly differ between groups.

### Color category analysis

Our goal was to test whether shape ordinality maps onto the six stages of entry into languages of color terms described by Berlin and Kay (1969). To do so, the CIELab values were converted into Berlin–Kay color categories (Berlin and Kay, 1969) using the online application Colournamer (Mylonas et al., 2010, 2013), a computation model trained by over 3,000 participants, which returns for color inputs the most likely color name in several languages and its Berlin–Kay color category in English. This method of converting 3D color space coordinates into Berlin–Kay color categories has been used in the recent studies assessing typical grapheme–color associations (Root et al., 2017, 2019; Rouw and Root, 2019). Upon visual inspection, the algorithm appears less precise for colors close to the grayscale axis (at a saturation below 1). Those color responses have therefore been corrected manually (629 data points, corresponding to 1.7% of the data).

The shape order has been numbered from 1 to 5, to denote their increasing number of sides, called complexity in the remainder of the analysis (circle:1; triangle: 2; square: 3; pentagon: 4; hexagon: 5).

#### Entry into languages order

A Spearman's Rho correlation analysis was run on the shape complexity as a function of color name entry into language order (Berlin and Kay, 1969). A correlation was found between entry into language order and shape complexity (ρ = 0.17, *p* < 0.001). A tendency for shape complexity order to follow color name entry into language order seems to appear.

#### Easy of generation order

Battig and Montague (1969) have shown that color name generation was not random, and that it followed a specific order. It could be possible therefore that less complex shapes are associated with easily generated color names, rather than names that enter first into the language. We, therefore, tested whether shape–color associations reflect shape complexity order being mapped from color name ease of generation order. A Spearman's Rho correlation analysis was run on the shape complexity order as a function of color name ease of generation order (Battig and Montague, 1969), following Simner et al. (2005)'s procedure. No correlation was found (ρ = 0.006, *p* = 0.175) between shape complexity and color category ease of generation order.

#### Color name frequency order

All color names do not have the same frequency in language. To investigate whether shape–color associations reflect shape complexity order being mapped from color name frequency order in language, color name frequencies in Danish were established based on the DaTenTen20 corpus (Danish web corpus) containing frequencies per 3.4 billion words. The Danish color names being inflected depending on the noun following them, the search was done on the root, gender-inflected, number-inflected, and determination-inflected variations of the eleven color categories from Berlin and Kay (1969). The resulting order can be found in Table 3. A Spearman's Rho correlation analysis was run on the shape complexity order, as a function of color name frequency order. A correlation was found between color name frequency order and shape complexity (ρ = 0.057, *p* < 0.001). These results suggested a slight tendency for shape complexity order to follow color name frequency.

**Table 3:** The 11 color categories ranked by ease of generation, color name frequency, and entry into human languages orders, in descending order.

**Ease of generation (Battig and Montague, 1969)**	**Color name frequency (DaTenTen20 corpus)**	**Entry into languages (Berlin and Kay, 1969)**
Blue	Black	Black/white
Red	White	Red
Green	Green	Green/yellow
Yellow	Red	Blue
Orange	Blue	Brown
Black	Yellow	Orange/purple/gray/pink
Purple	Gray	
White	Brown	
Pink	Orange	
Brown	Pink	
Gray	Purple	

Upon inspection, color name frequency order and entry into language order seem to resemble each other. A Spearman's Rho correlation analysis was run on the entry into language order, as a function of color name frequency order. A strong correlation was found between color name frequency order and entry into language order (ρ = 0.753, *p* < 0.001). These results suggested that the slight tendency for shape complexity order to follow color name frequency may be driven by the fact that it also follows the entry into language order tightly.

**Figure 10:**
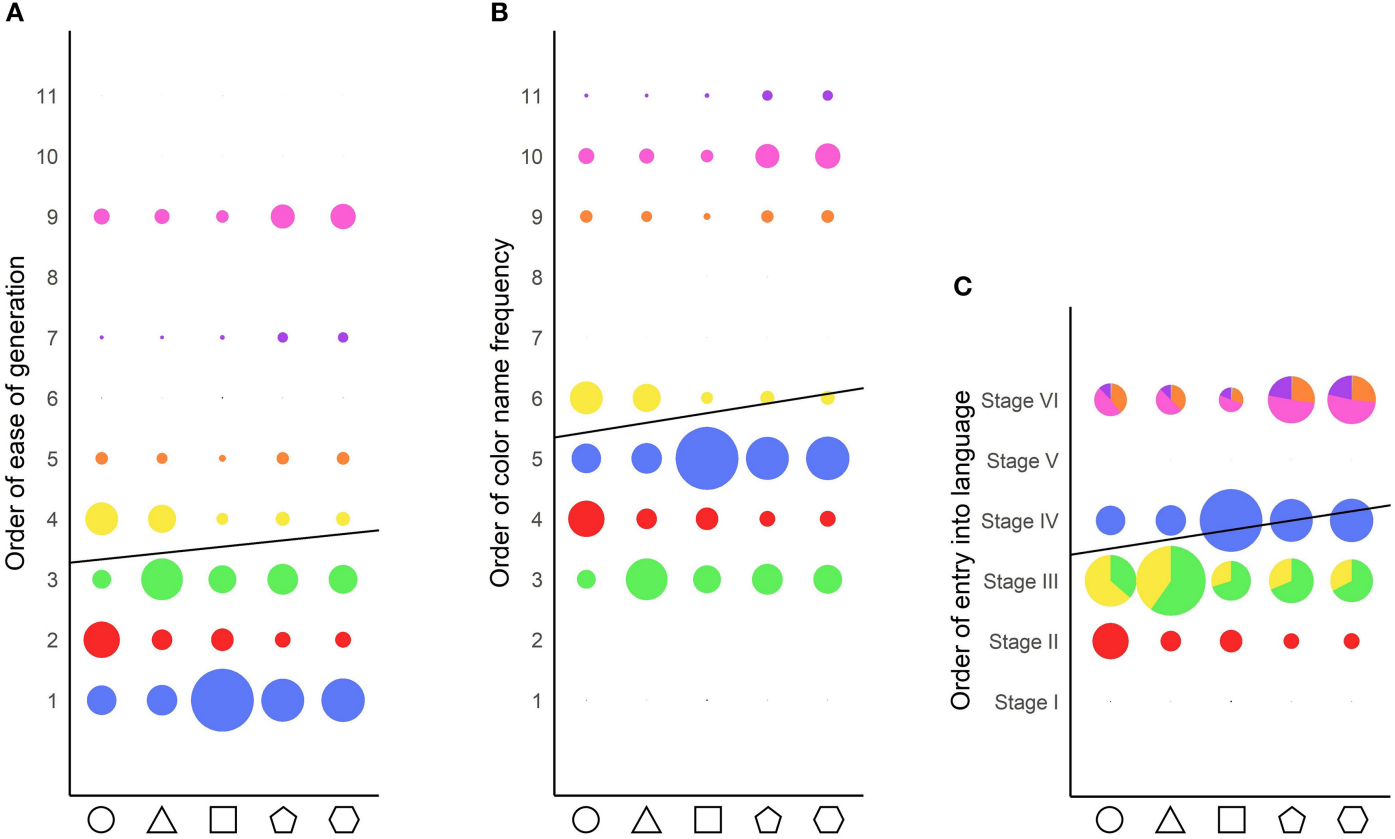
Correlation between shape order and the various color orders. The dot sizes reflect the frequency of response of each color category for each shape. The solid black line represents the correlation line. The colors depicted are the average color chosen by the participants for each color category. **(A)** Correlation of shape order with color name ease of generation order. **(B)** Correlation of shape order with color name frequency order. **(C)** Correlation of shape order with color name entry into language order. For stages containing more than one color category, the proportion of responses for each color category is denoted as the proportion of the color on the dots.

Figure 10 shows the correlation between shape order and the ease of generation (left panel), color name frequency (center panel), and entry into language (right panel) orders.

## Discussion

A large group of museum visitors have been asked to report their color intuitions regarding five shapes, using a full color wheel. The data were analyzed in terms of hue, saturation, and lightness, as well as color as a whole. Overall, Danish participants associated the circle more often with red and yellow, and preferred high lightness values. The triangle was associated with green and yellow, and featured high saturation and lightness values. The square was associated with the color blue and showed low lightness values. Finally, both the pentagon and hexagon were associated with magenta, as well as cyan, purple, and orange. These results indicate several points, first and foremost that the associations are partially in line with those presented in the past literature (Jacobsen, 2002; Jacobsen and Wolsdorff, 2007; Kharkhurin, 2012; Albertazzi et al., 2013; Makin and Wuerger, 2013; Chen et al., 2015a,b; Dreksler and Spence, 2019; see Table 1), where a tendency for red/yellow circle, yellow/red triangle, and blue/red square appeared. Second, despite those similarities to previous studies, the shape–color associations reported by Danish visitors do not fully match them. Notably, the association between green and triangle had never been attested before. It also goes against the general pattern of previous studies which saw the triangle being associated with yellow and red, red being the opponent color to green (Kay and McDaniel, 1978; Abramov and Gordon, 1994; Valberg, 2001; Witzel and Franklin, 2014). This variation could be due to cultural and/or methodological differences. Green was indeed not offered as a color chip response by Jacobsen (2002), who found a preference for the triangle-red association. There were, however, an equal number of yellow, red, and green color chips in Dreksler and Spence (2019)'s paradigm, which nevertheless led to associations with red and yellow for the triangle. The triangle–green association may thus be a Danish particularity. Transposing the full color wheel paradigm in a different culture would help clarify this result.

Our color wheel paradigm allowed us to unveil a pattern regarding saturation never attested before due to constraints from previous methodologies. Indeed the saturation property of color choices was always controlled for, and color options were always presented at full saturation in previous studies (Albertazzi et al., 2013; Chen et al., 2015a; Dreksler and Spence, 2019). When saturation is allowed to be manipulated by participants, we observe that significant shape–hue associations are more saturated than non-significant ones for the circle, triangle, and square, while no difference occurs for the pentagon and hexagon.^1^ Saturation can be defined as “how colorful a color is”. The triangle overall produced more saturated colors than the four other shapes, but when looking at only significant shape–hue associations, the circle, triangle, and square showed similar mean saturations, despite all having different significant hue associations. Interestingly, highly saturated colors have been shown to be preferred to those of low and mid saturations (Palmer and Schloss, 2010) and to produce higher arousal levels (Wilms and Oberfeld, 2018). Saturation levels could therefore reflect the strength of the association at the group level. This coincides with the fact that the circle, triangle, and square have also stronger biases toward certain hues compared to the pentagon and hexagon, as shown by the residual values in Figures 5, 9. On and on, our data indicate that the shape–color associations are more clearly settled for the circle, triangle, and square than for the pentagon and hexagon.

### The involvement of prototype categories

As described above, the circle, triangle, and square seem to be subjected to stronger shape–color associations than the pentagon and hexagon. Moreover, while the circle, triangle, and square each exhibit distinct color associations, the pentagon and hexagon are barely different from each other.

Our stimuli list, despite being small, had the particularity of containing only shapes that represent known geometrical concepts. More precisely, contrary to most recent studies on shape–color associations that featured a larger list of stimuli (Albertazzi et al., 2013; Chen et al., 2015a; Dreksler and Spence, 2019), our stimuli had the property of all being concepts with a precise name. However, those five shapes are not concepts participants are equally familiar with. The circle, equilateral triangle, and square are, according to the Gestalt psychology principle of “good form,” prototypes of shape categories (Rosch, 1973) and are therefore described as basic shapes. They differ from all other shapes (e.g., the pentagon, hexagon, or oval), which do not constitute prototype shape categories. In our study, we observe that the three prototypes of shape categories (circle, triangle, and square) are associated with the four primary color categories [red, blue, green, and yellow (Kay and McDaniel, 1978; Abramov and Gordon, 1994; Valberg, 2001; Witzel and Franklin, 2014)], whereas the non-prototypical shape categories (pentagon and hexagon) are associated with secondary color categories [namely, pink, purple, brown, and orange (Witzel and Franklin, 2014)]. This pattern seems very consistent across studies and cultures (Albertazzi et al., 2013; Chen et al., 2015a). The three prototypes of shape categories are, however, not randomly associated with the four primary color categories. They follow a pattern that is better explained by the stages of entry into languages (Berlin and Kay, 1969). A similar tendency has been observed in the general population for numbers and days of the week (Shanon, 1982), English and Arabic numbers and letters (van Leeuwen et al., 2016), and in synesthetes for letter frequency (Simner et al., 2005). Both intrinsic ordering and frequency have therefore been shown to map onto the Berlin and Kay (1969) stages of entry into languages. It is, however, not possible to determine whether it is intrinsic ordering or frequency that is at play regarding our shape stimuli, as they follow the same pattern, based on complexity order as measured by number of sides (Circle: 0; triangle: 3; square: 4) or frequency in language [Circle: 73,033; triangle: 36,773; square: 26,491/per 3.4 billion words (DaTenTen20)].

Past studies have hypothesized that grapheme–color associations derive from shape–color associations (Spector and Maurer, 2008, 2011) where toddlers tend to associate O and I with white but X and Z with black. Although synesthesia is highly idiosyncratic, synesthetes have been found to show a bias toward associating the letter O with the color white (Rich et al., 2005; Simner et al., 2005; Witthoft et al., 2015), and non-synesthetes have been found to associate the letter O to orange instead (Rich et al., 2005; Simner et al., 2005; Mankin and Simner, 2017). Non-synesthetes seem, therefore, to form letter–color associations based on phonology (i.e., O for orange) when possible. This has been witnessed for other initial letters of color words in adults (Rich et al., 2005; Simner et al., 2005; Spector and Maurer, 2011), and in children as early as from 7 years old (Spector and Maurer, 2008), but not in pre-literate children (Spector and Maurer, 2011) who based they letter–color associations on the visual properties of the letter O, and not on the sound of the letter neither. In our current study, participants were all above 6 years of age and significantly associated the circle with red and yellow. Furthermore, despite having 551 participants between 6 and 10 years of age, none of them associated the circle with white (i.e., the youngest participant who chose white for the circle was 13 years old). Interestingly, 19 of them associated the circle with orange. Our cluster analysis per age group showed that the older group (i.e., 35–100 years) significantly associated orange with the circle on top of red and yellow but not the younger (i.e., 6–34 years) group. Spector and Maurer (2008) found a significant association between the letter O and white in 7- to 9-year-old children and adults. However, it is important to note that the stimuli were only white and black and aimed, therefore, to probe preference for only those two colors. This means that for all age groups, the results only allowed to conclude that the letter O fits the color white more than it fits the color black. In other words, their paradigm did not allow to investigate other color associations to the letter O, such as red, yellow, orange, blue, etc. In line with our data indicating involvement of the conceptual identity of the stimuli, it is a possibility that interpreting the circle stimuli as a geometrical shape leads to color associations (i.e., red or yellow) that differ from the color associations that would be reported if the stimuli had been interpreted as a grapheme. It is also a possibility that the older group interpreted the circle stimuli as a letter more often than the younger group did, but our data do not allow us to evaluate this hypothesis. Furthermore, it would be interesting to replicate Spector and Maurer (2008)'s findings regarding associations with the letter O in pre-literate children in a paradigm offering more color options. Indeed, the circle in our data was significantly associated with colors at Stages II and III of entry into language, while the color white belongs to Stage I. Further research could elucidate whether in toddlers, who do not yet have a conceptual knowledge of shapes (Clements et al., 1999, 2018; Clements and Sarama, 2000, 2020), shape associations occur only for colors forming the Stage I of entry into language, and associations with colors at higher stages are developed as higher-level conceptual knowledge are formed.

### Comparison with other mediating factors previously proposed

Shape–color associations have been proposed to be mediated by perceived temperatures (Albertazzi et al., 2013; Chen et al., 2015a) or emotions (Malfatti et al., 2014). A strong shape–temperature/emotion–color association does, however, not inform us about the directionality of the associations. Shape–color associations may emerge based on the shapes' perceived temperature/emotion, which in turn triggers a color matching this temperature/emotion. However, conversely, it could be the shapes' perceived colors that trigger a feeling of temperature/emotion.

Our study differs from the ones previously cited because it contained only shapes that could be named [as opposed to Dreksler and Spence (2019) and Malfatti (2014)'s stimuli lists]. Our findings nevertheless do not necessarily negate the previously proposed mediating factors. Indeed, comparing our study's results with Malfatti (2014) and Dreksler and Spence (2019) may be a case of comparing apples and oranges. In the same way as colors are low-level perceptual entities that can be decomposed into hue, lightness, and saturation, shapes can be decomposed into symmetry, angularity, and complexity. However, colors are also high-level mental categories, with names and prototypical members (Berlin and Kay, 1969; Rosch, 1973). Similarly, shapes also form high-level mental categories, with clear definitions and names (Rosch, 1973). As such, our study is more interested in shapes as high-level and learned categories, than as low-level perceptual properties. This distinction between shapes as perceptual information and conceptual information may be central to understanding the variety of results in the shape–color literature. As an example, Albertazzi et al. (2015) found when looking only at the angle aspect of shapes that 22.5° angles were associated with yellow, 45°, 90°, and 135° angles to green and yellow, and 157.5° angles to red and blue. However, they had previously found that the square (90° angles) was associated to red and blue (Albertazzi et al., 2013). This discrepancy could be due to looking at, on the one hand, low-level perceptual information (i.e., angles and symmetry), and on the other hand, at high-level concepts (shape categories).

### Limitations

The current study featured a limited number of stimuli, due to the museum context requirements. It would be interesting in the future to offer a greater variety of shapes, including, for example, an inverted triangle, equilateral diamond, isosceles triangle, rectangle, and oval, which would allow for testing the weight of perceptual (square vs. equilateral diamond) and categorical (square vs. rectangle) differences on shape–color associations.

The museum setup, although offering the chance to test a high number of visitors, was not as controlled as a lab environment. Several factors could have at times influenced the color decisions of the visitors, such as background noises (Ward et al., 2006; Hamilton-Fletcher et al., 2017), subtle lightness changes, or surrounding odors (Gilbert et al., 1996; Schifferstein and Tanudjaja, 2004; Demattè et al., 2006a). Furthermore, visitors could have been influenced by discussions with other visitors. However, our high number of participants should allow to diminish the impact of such noise in our data. The experiment probing shape–color associations was run on only one computer, diminishing the possibility of simply copying other visitors' answers.

Our sample also featured twice more women than men. Color-related gender difference has been found at the perceptual (Bimler et al., 2004), categorization (Sørensen and Kyllingsbæk, 2012), and linguistics (Simpson and Tarrant, 1991; Greene and Gynther, 1995; Mylonas et al., 2014; Fider and Komarova, 2019) levels. It would, therefore, be interesting to investigate shape–color associations in a gender-balanced paradigm.

Finally, our study focuses on only one culture. The same paradigm could be transposed to a different cultural population.

## Data availability statement

The original contributions presented in the study are included in the article/Supplementary material, further inquiries can be directed to the corresponding author.

## Ethics statement

Ethical approval was not provided for this study on human participants because the study falls under the Danish National Videnskabsetisk Komité Law (§ 14, part 2), by which behavioral studies not involving biological material are exempted from regional Ethics Committee. Written informed consent to participate in this study was provided by the participants' legal guardian/next of kin.

## Author contributions

TAS and AZ contributed to the study design and data collection. AZ performed the testing and data analysis and drafted the manuscript. AZ did the interpretation under the supervision of TAS and XL. TAS and XL provided critical revisions. All authors approved the final version of the manuscript for submission.

## Footnotes

^1^It is important to mention that, on the color wheel, less saturated colors were closer to the center of the wheel, and more saturated ones, toward the outskirt. No saturation bar needed to be manipulated to affect saturation, making the highly saturated and less saturated colors equally reachable by one single press on the wheel. This was not the case with lightness, which could only be manipulated by using the lightness bar, thus involving more interactions with the screen.
