# Supplementary material for: Shape–color associations in an unrestricted color choice paradigm
Source: Front Psychol. 2023 Jun 2;14:1129903. doi: 10.3389/fpsyg.2023.1129903 (PMC10273845; doi:10.3389/fpsyg.2023.1129903)
Supplement: Supplementary file 1 [file Table_1.docx]

|  | **Cluster** | **L** | **a** | **b** | **Color name** | **Frequency** |
| --- | --- | --- | --- | --- | --- | --- |
| Circle | 1 | 97.66 | -0.94 | 0.86 | White | 133 |
|  | 2 | 88.09 | -4.36 | 81.17 | Yellow | 1661 |
|  | 3 | 55.35 | 74.69 | 54.32 | Red | 1733 |
|  | 4 | 38.86 | 65.25 | -96.86 | Blue | 632 |
|  | 5 | 61.85 | 86.55 | -45.25 | Magenta | 252 |
|  | 6 | 88.49 | -78.35 | 74.36 | Green | 453 |
|  | 7 | 89.91 | -43.84 | -11.99 | Cyan | 195 |
| Triangle | 1 | 88.61 | -77.57 | 73.05 | Green | 1318 |
|  | 2 | 55.27 | 75.11 | 54.87 | Red | 913 |
|  | 3 | 90.33 | -45.23 | -9.86 | Cyan | 257 |
|  | 4 | 40.45 | 62.07 | -94.23 | Blue | 716 |
|  | 5 | 92.54 | -14.59 | 85.05 | Yellow | 1172 |
|  | 6 | 61.8 | 86.77 | -47.66 | Magenta | 315 |
|  | 7 | 69.19 | 38.09 | 69.59 | Orange | 273 |
| Square | 1 | 43.18 | 55.27 | -89.75 | Blue | 2349 |
|  | 2 | 94.42 | -17.66 | 86.19 | Yellow | 344 |
|  | 3 | 54.71 | 76.1 | 56.04 | Red | 957 |
|  | 4 | 88.52 | -78.46 | 73.08 | Green | 774 |
|  | 5 | 90.76 | -45.96 | -9.67 | Cyan | 196 |
|  | 6 | 1.11 | 0.01 | -0.27 | Black | 74 |
|  | 7 | 97.66 | -0.02 | 0.71 | White | 88 |
|  | 8 | 63.02 | 82.92 | -42.01 | Magenta | 93 |
|  | 9 | 85.52 | -33.87 | -22.5 | Light Blue | 39 |
| Pentagon | 1 | 55.06 | 75.24 | 56.43 | Red | 602 |
|  | 2 | 43.22 | 55.63 | -89.7 | Blue | 1185 |
|  | 3 | 94.17 | -18.11 | 86.21 | Yellow | 374 |
|  | 4 | 88.58 | -77.45 | 71.57 | Green | 852 |
|  | 5 | 57.86 | 85.32 | -55.53 | Magenta | 877 |
|  | 6 | 69.77 | 36.84 | 69.52 | Orange | 354 |
|  | 7 | 87.53 | -39.6 | -15.34 | Cyan | 496 |
|  | 8 | 70.43 | -7.76 | -45.8 | Light Blue | 51 |
| Hexagon | 1 | 60.69 | 60.45 | 61.3 | Red | 1007 |
|  | 2 | 88.92 | -43.54 | -11.42 | Cyan | 476 |
|  | 3 | 88.57 | -77.66 | 72.34 | Green | 755 |
|  | 4 | 43.06 | 56.26 | -89.85 | Blue | 1180 |
|  | 5 | 58.24 | 85.88 | -54.93 | Magenta | 894 |
|  | 6 | 93.72 | -17.53 | 85.37 | Yellow | 356 |
|  | 7 | 65.39 | 1.81 | -53.74 | Light Blue | 105 |
|  | 8 | 79.18 | 14.57 | 76.56 | Orange | 39 |

**Table S1. Mean CIELab coordinate values for the clusters resulting from the clustering analysis of each shape, along with their frequency count and color name.**
